# Iron Oxide Nanoparticles in Mesenchymal Stem Cell Detection and Therapy

**DOI:** 10.1007/s12015-022-10343-x

**Published:** 2022-02-01

**Authors:** Kosha J. Mehta

**Affiliations:** Centre for Education, Faculty of Life Sciences and Medicine, King’s College London, London, UK

**Keywords:** Mesenchymal stem cells, iron, iron oxide nanoparticles, MSC therapy, MSC detection, Stem cell therapy

## Abstract

Mesenchymal stem cells (MSCs) exhibit regenerative and reparative properties. However, most MSC-related studies remain to be translated for regular clinical usage, partly due to challenges in pre-transplantation cell labelling and post-transplantation cell tracking. Amidst this, there are growing concerns over the toxicity of commonly used gadolinium-based contrast agents that mediate in-vivo cell detection via MRI. This urges to search for equally effective but less toxic alternatives that would facilitate and enhance MSC detection post-administration and provide therapeutic benefits in-vivo. MSCs labelled with iron oxide nanoparticles (IONPs) have shown promising results in-vitro and in-vivo. Thus, it would be useful to revisit these studies before inventing new labelling approaches. Aiming to inform regenerative medicine and augment clinical applications of IONP-labelled MSCs, this review collates and critically evaluates the utility of IONPs in enhancing MSC detection and therapeutics. It explains the rationale, principle, and advantages of labelling MSCs with IONPs, and describes IONP-induced intracellular alterations and consequent cellular manifestations. By exemplifying clinical pathologies, it examines contextual in-vitro, animal, and clinical studies that used IONP-labelled bone marrow-, umbilical cord-, adipose tissue- and dental pulp-derived MSCs. It compiles and discusses studies involving MSC-labelling of IONPs in combinations with carbohydrates (Venofer, ferumoxytol, dextran, glucosamine), non-carbohydrate polymers [poly(L-lysine), poly(lactide-co-glycolide), poly(L-lactide), polydopamine], elements (ruthenium, selenium, gold, zinc), compounds/stains (silica, polyethylene glycol, fluorophore, rhodamine B, DAPI, Prussian blue), DNA, Fibroblast growth Factor-2 and the drug doxorubicin. Furthermore, IONP-labelling of MSC exosomes is reviewed. Also, limitations of IONP-labelling are addressed and methods of tackling those challenges are suggested.

**Graphical Abstract:**

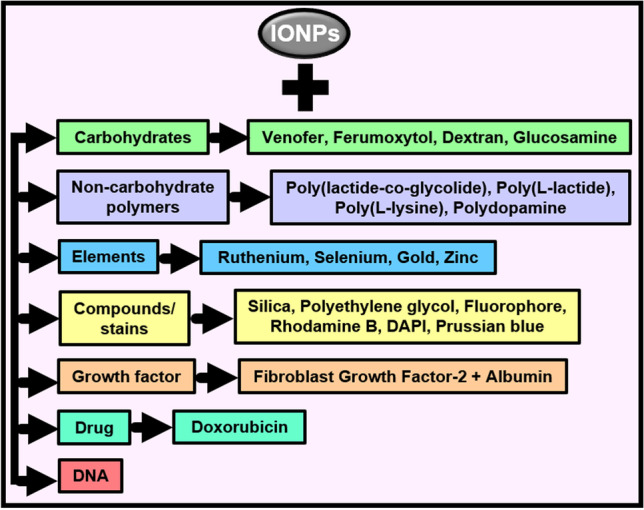

## Introduction

Mesenchymal stem cells (MSCs) are present throughout the human body in various tissues. MSCs show niche-dependent multilineage differentiation and secrete therapeutic exosomes and growth factors (paracrine effects) to support various physiological processes. These cells can migrate to sites of injury/inflammation and demonstrate their regenerative and reparative properties. Also, MSCs exhibit tropism towards tumours. Thus, MSCs possess the ability to facilitate both cell-based and cell-free therapeutics and promise targeted therapy [1–4].

MSCs are usually extracted from bone marrow, adipose tissue, umbilical cord, and placenta. These cells are easy to isolate, the cells expand in-vitro, retain phenotypic characteristics and differentiation potential in-vitro, and make autologous transplantation feasible [5, 6]. MSCs offer advantages over other transplantable stem cells such as embryonic and induced pluripotent stem cells and are therefore gaining popularity. Allogenic MSC transplantation has been performed on patients with haematological malignancy, cardiomyopathies and graft v/s host disease [6]. Promising results have been obtained in clinical trials that targeted amelioration of knee osteoarthritis [7, 8], multiple sclerosis [9] and spinal cord injury [10], as well as in animal models of myocardial infarction [11–13], multiple sclerosis [14], sensorineural hearing loss [15], and bone defects [16]. Moreover, using MSCs to treat COVID-19 patients has been discussed [17–20].

## Rationale

Hitherto, the therapeutic potential of MSCs has been assessed in over a thousand MSC-related clinical trials, as listed on the Clinical Trials Register for EU (https://www.clinicaltrialsregister.eu/) and ClinicalTrials.gov for USA (http://clinicaltrials.gov/). However, only a few have been translated successfully into regular clinical practice [6]. While this is partly due to the unavailability of sufficient number of MSCs for clinical utility [21] and the risks and side-effects posed by MSCs post transplantation [22, 23], this is also due to the issues encountered during pre-administration labelling procedures that subsequently affect MSC detection, homing, and therapeutic potential. Notably, the healing benefit of MSCs is dependent on their ability of homing at target sites [23] where these cells secrete trophic and immunomodulatory molecules [3]. This raises the significance of pre-transplantation labelling procedures to achieve maximum therapeutic efficiency. Moreover, the transplanted MSCs must effectively differentiate at the target site to execute their regenerative function, but MSC differentiation ability in-vivo could be low. For successful homing and differentiation in-vivo, the pre-administration approaches require multi-step and complex in-vitro methodology, which is yet to be perfected. These aspects collectively challenge the detection and retention of transplanted MSCs and thereby hamper their frequent clinical usage.

Prior to transplantation, MSCs are often labelled with contrast agents to facilitate subsequent detection and tracking of cells via magnetic resonance imaging (MRI). Although paramagnetic ions involving chromium, manganese, and gadolinium are commonly used as MRI contrast agents, there are growing concerns over their toxicity; particularly, gadolinium-based contrast agents that have been used in pre-clinical models [24]. Also, gadolinium exposure may contribute to nephrogenic systemic fibrosis in patients with reduced kidney function and may cause gadolinium deposition in the brain in patients with normal kidney function [25]. Moreover, most agents distribute systemically and therefore show poor performance in certain clinical applications, for example, gastrointestinal imaging [26].

Thus, it is essential to search for effective but less toxic substitutes that would retain the ability to detect the transplanted MSCs non-invasively, preserve MSC homing and therapeutic potential, and enhance their reparative properties post transplantation.

MSCs can be labelled with iron oxide nanoparticles (IONPs) that act as good MRI contrast agents [27]. The imaging artifact posed by IONPs is much larger than the labelled cell. So, in this context, these are superior to gadolinium-based contrast agents [28]. IONP-labelling of MSCs has been tested in-vitro, and labelled MSCs have been successfully detected by MRI and tested in animal models of disease [29], where the combination of IONP-labelling of MSCs and MRI has shown huge diagnostic and therapeutic potential [30]. Therefore, before reinventing the wheel and exploring new approaches, it would be beneficial to revisit the field and assess IONP’s potential in improving MSC detection and homing post-administration. Then, these approaches could be modified to augment MSC therapy for future clinical applications.

Accordingly, this review conglomerates and critically evaluates the usage of IONPs in enhancing MSC detection and therapeutics. By exemplifying several clinical pathologies, it examines MSC-labelling of IONPs in combination with carbohydrates, non-carbohydrate polymers, elements, compounds, stains, DNA, growth factor, and the drug doxorubicin. Limitations are mentioned and putative solutions are suggested.

## Fundamentals of Labelling MSCs with IONPs

The success of any cell therapy is dependent on the migration, engraftment, homing, proliferation, and differentiation of the transplanted stem cells at the target site where these cells exhibit their reparative functions. With regard to MSCs, there is loss of transplanted MSCs in-vivo and there is only little evidence that the MSCs proliferate in-vivo post transplantation [6]. Thus, it is important to longitudinally track the destination and fate of the transplanted MSCs. This not only helps evaluate cell treatment efficacy but also aids in formulating and improvising the time, dosage, and delivery route of cell-transplants.

Post-transplantation cell engraftment can be detected through histological analysis of sacrificed animals or tissues. This is invasive and does not facilitate long-term monitoring of transplanted cells in humans. Thus, non-invasive methods are used, which include the usage of endogenous molecules with intrinsic fluorescence or external fluorescent and non-fluorescent molecules that can be tracked by in-vivo imaging. However, these contrast agents are challenged by low transfection efficiency, photo-bleaching over time, in-vivo degradation and interference from tissue autofluorescence [27]. Therefore, alternative approaches of MSC labelling are sought that provide reliable real-time cell tracking non-invasively.

MRI is a non-invasive method for cell tracking. It involves exposure of cells and tissues to high magnetic fields, which enables visualisation of the transplanted cells in-vivo. It has varied clinical applications and is more popular than other methods because it offers deep penetration, 3-D imaging, better spatial resolution and soft tissue contrast without ionising radiation [26]. It is not possible to directly distinguish between host cells and transplanted cells on a cellular scale using the usual MRI resolution. Therefore, cells to be transplanted are labelled with MRI-visible particles i.e., contrast agents like IONPs to increase the resolution and contrast of the image so that areas of interest can be easily identified.

**Fig. 1:**
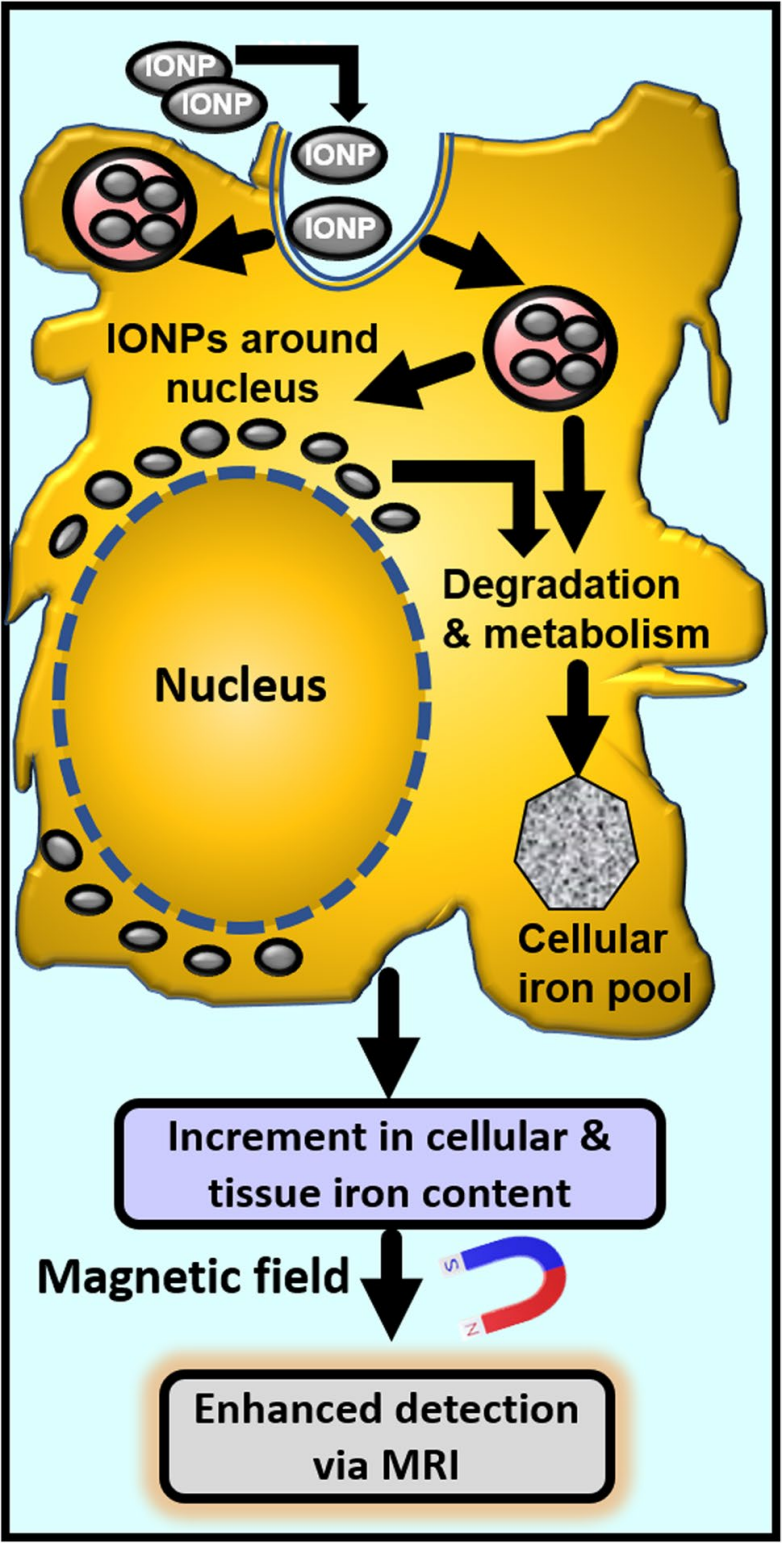
Iron oxide nanoparticle (IONP) endocytosis and enhanced detection via MRI. IONPs can be taken up into endo/lysosomes by receptor-mediated endocytosis, phagocytosis, or pinocytosis. A clathrin-mediated and actin-dependent endocytosis of IONPs in MSCs has been reported. IONPs localise within lysosomes and around the nucleus. After degradation and metabolism, iron inside these particles is incorporated into the cellular iron pool. Total cellular/tissue iron content is increased, which facilitates detection via MRI [27, 28, 30, 33]

MRI has been used for detecting hepatic iron in transfusion iron-overload [31, 32], where the paramagnetic effect of hemosiderin [water-insoluble, degraded ferritin (iron)] generates local field inhomogeneities such that the regions where iron is present appear darker than other regions in the field. MSC labelling with IONPs is based on this principle. Essentially, entry of IONPs inside the cells increases cellular/tissue iron content (Fig. 1). Upon exposure to a magnetic field, these labelled cells/tissues create and increase localised inhomogeneities in the magnetic field by inducing a negative contrast i.e., a darker area (spots) in regions where iron is accrued. This increases the visual contrast between different tissues and thereby aids in cell visualisation and tracing of transplanted cells in-vivo via MRI. IONPs have been used for clinical imaging for more than two decades [34].

Typical structural and functional characteristics of IONPs have been tabulated (Table 1). Also, some features of IONP-labelling of MSCs have been tabulated (Table 2).

**Table 1:** Selected structural and functional characteristics of IONPs

Parameter	Characteristics of IONPs
Composition	IONPs are composed of a bioreactive iron oxide core (either magnetite Fe_3_O_4_ or maghemite *γ*-Fe_2_O_3_) surrounded by biologically compatible ligands. • Core: contains thousands of iron atoms that collectively increase localiron concentration enabling the detection of low numbers of cell populations that contain these particles. • Ligands: include silica, citrate, dextran or carboxydextran, chitosan, gelatin or starch that generally prevent particle aggregation and provide hydrophilicity and stability to the internal magnetic core [27, 35]. Usually, IONPs with a particle diameter > 50 nm are considered as superparamagnetic IONPs (SPIONPs) and those with diameter < 50 nm are considered as ultrasmall SPIONPs (USSPIONPs) [36].
Magnetism and colloidal stability	• Particles are colloidally stable [27, 37] • When exposed to a magnetic field, IONPs show superparamagnetism i.e., a strong response (magnetic properties) and thereby high sensitivity in detection via MRI. • Due to the magnetic properties, an external magnetic field can be used to direct IONPs to specific sites within the tissue, which facilitates targeted delivery of therapeutic agents [38]. • When the magnetic field is removed, IONPs lose their magnetisation vector, become highly dispersed, show no magnetism at room temperature. There is almost no particle self-aggregation and phagocytic uptake is prevented.
At physiological level	• Most IONPs are phagocytosed by the liver Kupffer cells. This promotes their utility for liver imaging [34]. • Generally, nanoparticles with less than 10 nm are eliminated via the renal system whereas those with size greater than 200 nm are phagocytosed. Both events are not beneficial for biomedical applications [37].
Biodegradability	IONPs remain in the circulation for a long time but are biodegradable in nature i.e., get cleared from the circulation by opsonins. Essentially, opsonins activate the complement system and tag the IONPs to be engulfed/degraded by the phagocytic cells [39]. However, inorganic non-biodegradable IONPs may remain in the environment for long periods, leading to prolonged exposure to human with unidentified consequences.
Half-life	• USSPIONPs have a longer half-life in blood than SPIONPs, so these cater to a wider spectrum of imaging application such as tumour perfusion imaging, atherosclerotic plaque imaging, MR angiography, and the imaging of liver, lymph node and bone marrow. • Also, USSIONPs are not linked with a risk of developing nephrogenic sclerosis, which is particularly useful for patients with renal insufficiency and the approach is considered much safer than using gadolinium chelates for MRI [34].

**Table 2:** Notable features of IONP-labelling of MSCs

Parameter	Features of IONP-labelling of MSCs
MSC viability	IONPs seem to be non-toxic and do not elicit other side-effects [40]. IONPs generally do not affect MSC viability, proliferation and differentiation potential [27]. However, the long-term effects of these on MSC functions are yet to be fully understood.
MSC differentiation	For successful therapy, the transplanted IONP-labelled MSCs must retain viability and differentiation capability in-vivo. IONP-labelling to human bone marrow-derived MSCs (BM-MSCs) showed retention of surface markers, MSC trilineage differentiation capacity, and ability to differentiate into cardiac and neuronal cell lineages in-vitro [41].
MSC migration	MSCs show migration to injury sites, but the efficiency is low [42]. In rats with inflamed ear, injection of IONP-labelled MSCs showed improved migration of labelled MSCs to the site of inflammation. Essentially, IONPs enhanced MSC migration to injury sites and facilitated healing, thereby showing the potential for improved clinical efficacy. This was also observed in-vitro wherein IONPs elevated MSC migration and homing ability (Fig. 2) [2].
Adipose-derived MSCs in context	Bone marrow is the most common source of MSCs for clinical use. Using adipose-derived MSCs is considered as a potential alternative. In-vitro, IONP-loaded adipose-derived stem cells retained their ability of multilineage differentiation and did not differ from unlabelled cells in expression levels of caspase-3, interleukins (IL)-6 and 8 and vascular endothelial growth factor (VEGF) for 4 weeks following IONP-labelling [38]. However, in-vitro, these cells showed variable differentiation depending on the type, duration and intensity of the magnetic field applied to these cells; shorter and longer exposure times with low intensity magnetic field promoted adipogenesis and osteogenesis, respectively [46]. Although adipose-derived stem cells have shown promising results including neurocognitive improvement in animal models [47], these cells can confer malignant features to cervical cancer cells [48]. Therefore, its clinical usage (with or without IONP-labelling) for tumour treatment needs further investigation.

## MSC Tropism towards Tumour/Inflammation and Contextual IONP-Induced MSC Alterations

**Fig. 2:**
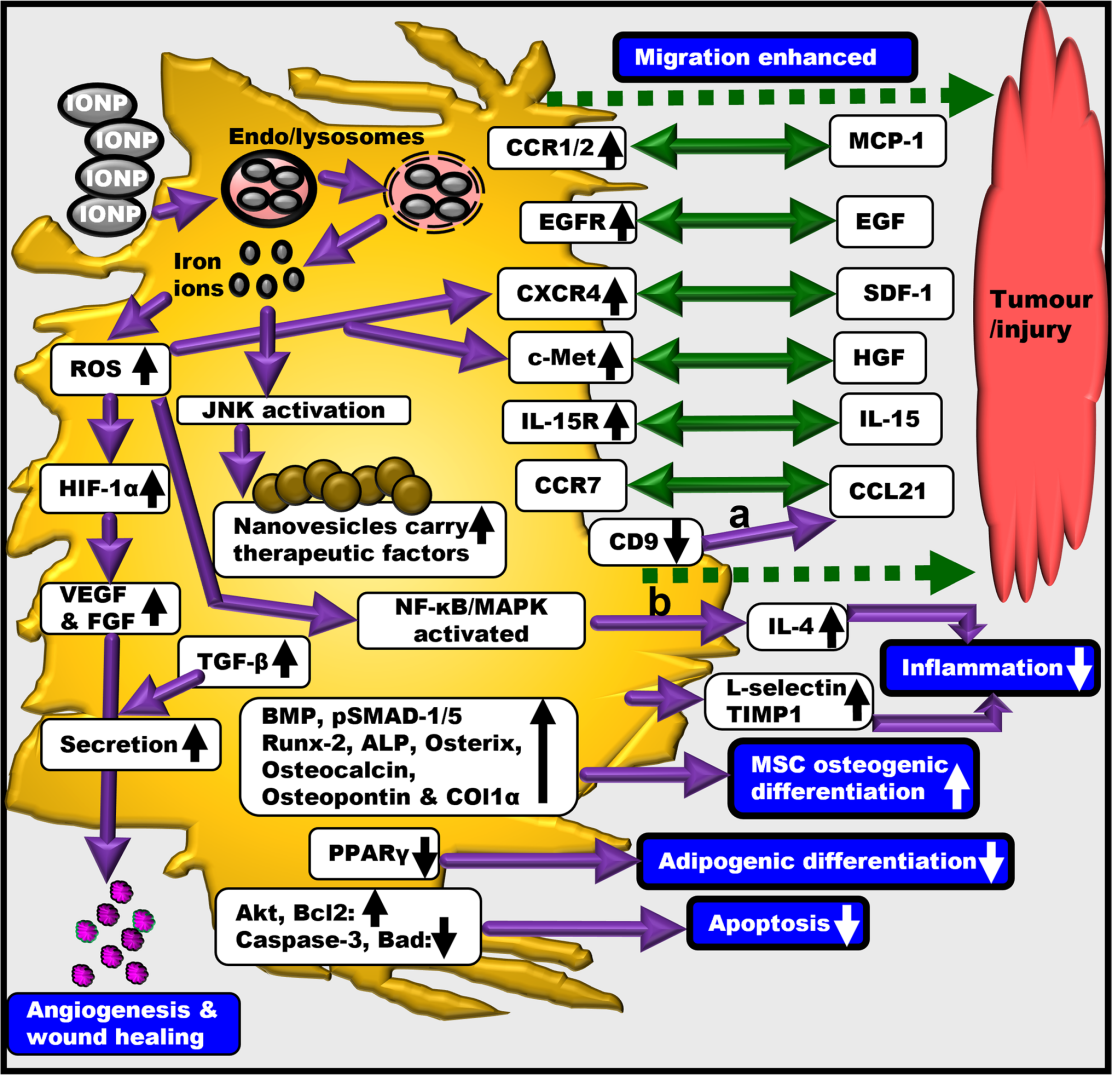
Iron oxide nanoparticle (IONP)-induced alterations in MSC biology. IONPs can cause several intracellular alterations within the MSCs leading to varied responses. Increased and decreased signalling/expression/effect is shown by upward and downward pointing arrows, respectively. Chemokine-receptor pairing has been shown via double-sided green arrows. Dotted green arrows indicate MSC tropism towards tumor/injury. Interestingly, both MSCs and tumour cells can secrete TGF-β and VEGF (a): IONP-induced reduction in CD9 expression may lead to unaltered CCL21 secretion by MSCs, thereby retaining the migration of tumour cell. The relevant study is discussed in the review [43]. (b): IONP-labelled MSCs produce increased levels of IL-4. IONPs can activate MAPK signalling in MSCs [44]. Activation of this pathway regulates the production of IL-4 and other cytokines. Genetically modified MSCs that secreted IL-4 (anti-inflammatory) in response to NFκB activation demonstrated great immunomodulatory ability and mitigated the pro-inflammatory response of macrophages [45]. ALP: Alkaline phosphatase; CCL21: C-C motif ligand-21; CXCR: C-X-C motif chemokine receptor; EGF: Epidermal growth factor; EGFR: Epidermal growth factor receptor; FGF: Fibroblast growth factor; HIF1-α: Hypoxia inducible factor 1 alpha; IL: Interleukin; MCP-1: Monocyte chemoattractant protein-1; PPARγ: Peroxisome proliferator-activated receptor gamma; SDF-1: Stromal cell-derived factor-1; TIMP-1: Tissue inhibitor of metalloproteinase 1; VEGF: vascular endothelial growth factor

MSCs exhibit tropism towards tumours [1] and have shown to home into tumours of animal models of cancers; namely, in cancers of liver, lung, breast, colon and brain glioma. This MSC tropism and homing involves chemokines released from tumours that attract and recruit MSCs with corresponding receptors (Fig. 2) [49]. For example, glioma cells release many chemokines and growth factors including stromal cell-derived factor-1 (SDF-1), monocyte chemoattractant protein-1 (MCP-1), transforming growth factor-beta (TGF-β), VEGF, IL-8 and neurotrophin-3. These chemokines support MSC tropism for gliomas [50] by pairing with receptors on MSCs (Fig. 2). Examples of cytokine-receptor pairs implicated in MSC tumour tropism are SDF-1 & C-X-C motif chemokine receptor-4 (CXCR4), VEGF & VEGFR, MCP-1 & C-C motif chemokine receptor (CCR2), epidermal growth factor (EGF) & EGFR, and hepatocyte growth factor (HGF) & c-Met [51]. These pairs have also been implicated in MSC tropism towards injury/inflammation sites (Fig. 2). However, in the MSCs, expression levels of receptors like CXCR4, CCR1 or c-met can be low. Therefore, several studies have been conducted to improve their expressions in MSCs to enhance MSC migration [2].

Injury-induced release of cytokines e.g. SDF-1 can upregulate CXCR4 on MSC surfaces and this can promote chemotaxis i.e. migration of regenerative cells and homing of MSCs into target sites [5, 52, 53]. Likewise, as shown in Fig. 2, IONPs can increase MSC expressions of CXCR4 and the migration-related proteins CCR1 and c-Met [2]. Essentially, intracellularly delivered iron ions (due to IONP treatment) induce low levels of ROS [54, 55], which can elevate CXCR4 in MSCs [55–57], amongst other MSC alterations (Fig. 2). Similarly, IONPs induce elevation in MSC EGFR and this supports MSC tumour tropism (towards tumour EGF) (Fig. 2) [58]. Iron-ion-induced ROS also upregulates hypoxia inducible factor-1α (HIF-1α) expression in MSCs, which in turn, can elevate MSC secretion of paracrine factors like VEGF (supports angiogenesis during wound repair) and basic fibroblast growth factor (bFGF) (supports angiogenesis) [59]. Essentially, HIF-1α acts as a transcription factor, binds to VEGF promoter and induces VEGF expression [56, 60, 61].

Although MSCs exhibit tropism towards malignant tumours and are therefore considered as vehicles for the delivery of therapeutic materials, the exact function of MSCs following homing into tumours remains to be fully elucidated. Studies have reported both pro-tumour and anti-tumour potential of MSCs [62]. The anti-tumour MSC mechanisms possibly involve inhibition of angiogenesis and Wnt, Akt and NFκB signalling, and increased cell cycle arrest and apoptosis [63], while MSC pro-tumour effects [5] involve promotion of tumour growth and metastasis, as observed in hepatocellular carcinoma in-vivo [64]. Notably, iron-loading has shown to elevate mesenchymal and metastatic markers in HepG2 cells [65]. Therefore, iron-labelling of MSCs can further increase the pro-tumour potential of the MSCs. Thus, this approach must be executed with extreme caution.

Essentially, IONP endocytosis by the MSCs causes several other intracellular alterations including activation of signalling pathways and increased or decreased production of specific proteins (Fig. 2). These lead to altered MSC responses that are manifested as enhanced MSC migration towards tumour or injury, reduced inflammation and apoptosis, promotion of MSC osteogenic differentiation and reduction in MSC adipogenic differentiation (Fig. 2). IONP-labelled MSCs can produce increased levels of IL-4 (Fig. 2) in addition to elevation in other anti-inflammatory chemokines, thereby facilitating reduction in inflammation. While both IONP-labelled and unlabelled MSCs can show increased expression of TGF-β expression that affects the wound healing process, serum levels of VEGF and anti-inflammatory cytokines IL-10 and IL-4 have been higher in rats treated with IONP-labelled MSCs than rats treated with unlabelled MSCs [57], thereby demonstrating the healing effect exerted by labelled MSCs (Fig. 2).

## IONPs in Combination with Carbohydrates for Labelling MSCs

### Venofer

Venofer is an Fe^3+^-hydroxide sucrose complex with Fe^3+^-hydroxide in the core surrounded by covalently-bounded sucrose molecules [66]. It is used as an infusion-treatment for patients with iron deficiency anaemia [67].

A healthy cartilage is made of chondrocytes and there are several inflammatory conditions that damage the cartilage tissue. Aiming to devise a therapeutic strategy, Venofer-labelled MSCs from patients undergoing spinal surgery were examined for viability and chondrocyte differentiation ability in-vitro. Positive results demonstrated the potential of this approach in cell tracing and checking the cellular distribution of transplanted MSCs in-vivo [68]. Following this, Venofer-labelled human BM-derived MSCs demonstrated MSC trilineage differentiation in-vitro (osteogenic, adipogenic and chondrogenic). When transplanted into lapine intervertebral discs in rabbits, these were successfully tracked after 3 months post transplantation [69]. Results showed the potential application of this approach in human MSC transplantation to tackle degenerate cartilaginous tissues. Accordingly, human MSCs were labelled with Venofer and transplanted into degenerated intervertebral discs in patients. Transplanted cells and their progeny were detectable for up to 8 months post transplantation, showed distribution in different parts of intervertebral discs and MSC differentiation into chondrocyte-like cells [70]. This proved the success of this approach in humans.

Since MSCs have the ability to migrate to wound/inflammation sites and tumours [1], the therapeutic potential of Venofer-labelled MSCs against tumours was assessed. In-vitro, Venofer-labelling to dental-pulp-derived MSCs (by using protamine sulphate and heparin) retained MSC viability, allowed osteogenic and chondrogenic differentiation but hindered adipogenic differentiation (Fig. 2) [67]. This can be expected because stimulation of osteogenesis can inhibit adipogenesis, but on the other hand this defies observations where excess iron has shown to favour adipogenesis over osteogenesis [3]. This indicates that both free iron (which generates ROS and affects cell signalling pathways) as well as iron bound to moieties like sucrose (as in this case) can determine MSC differentiation. Regardless, in-vitro, these cells released exosomes in the conditioned medium, which, in combination with 5-fluorocytosine, caused tumour cell death in a dose dependent manner and demonstrated great therapeutic capability. When applied intranasally in rats, labelled cells successfully migrated to intracerebral glioblastoma, thus proving the capability of this method in tumour tracking [67], thereby promising targeted therapy for tumours.

Magnetic hyperthermia involves heating of tissues (tumour) to a local temperature of about 40 to 50 °C using magnetic materials, which causes cellular changes resulting in apoptosis or necrosis [71]; a useful approach for cancer treatment. In a further study, Venofer-labelled MSCs were used to facilitate magnetic hyperthermia. Human MSCs expressing yeast cytosine deaminase:uracil phosphoribosyl transferase suicide fusion gene were labelled with Venofer. Labelled human MSCs released exosomes. In presence of prodrug 5-fluorocytosine, these exosomes caused tumour cell death. The MSC-released exosomes contained iron oxide that were efficiently endocytosed by the tumour cells and the treated tumour cells could be successfully ablated by magnetic hyperthermia [72]. Venofer labelling was also successfully tested in-vitro in adipose-derived MSCs for tracking via MRI [66].

### Ferumoxytol

Ferumoxytol/Feraheme™ was originally designed as a contrast agent for MR [34]. It is the only FDA approved agent for intravenous treatment of anaemia in human and the only approved IONP that is used currently. Structurally, it is a carbohydrate-coated IONP i.e., magnetic core surrounded by polyglucose sorbitol carboxymethylether (carboxymethyldextran). Its particle size is approximately 17–30 nm and has a molecular weight of 750 kDa [73].

**Fig. 3:**
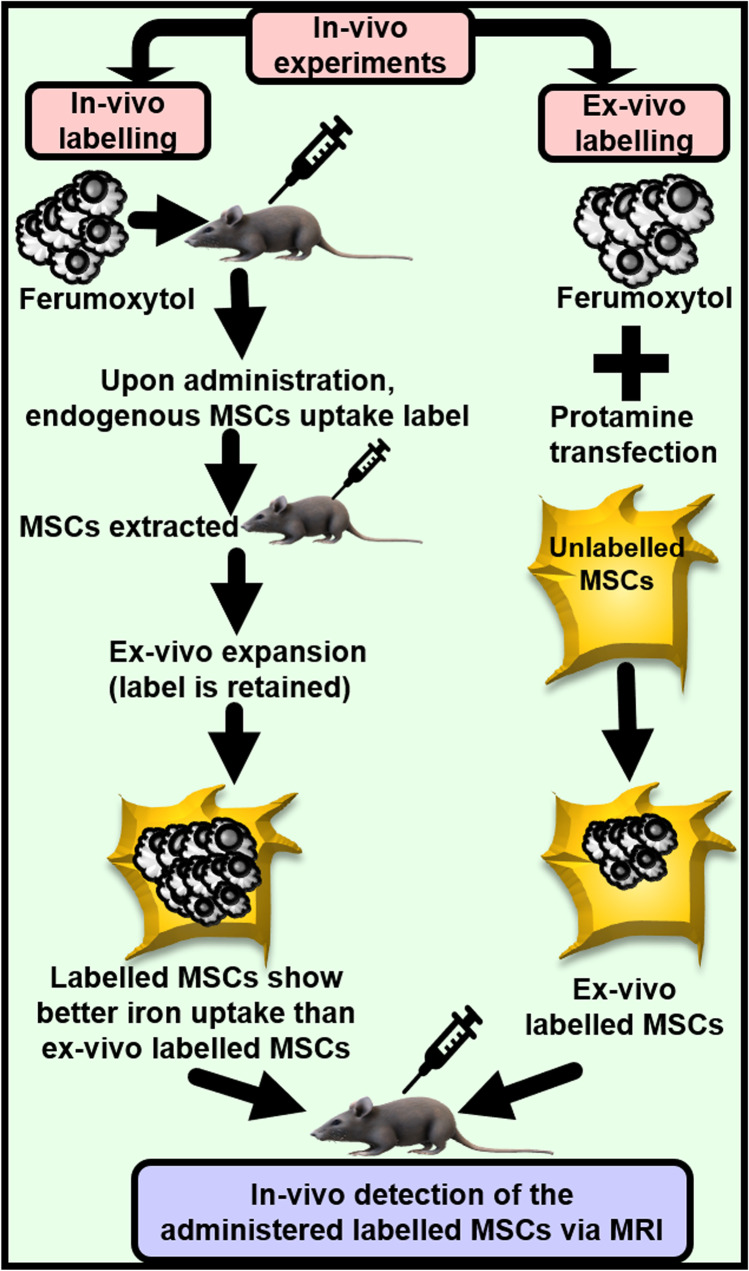
In-vivo and ex-vivo approaches of labelling MSCs with ferumoxytol**.** This figure is based on the elegant work done by Khurana et al. (2013). Prior to administration, MSCs could be labelled with IONPs ex-vivo or in-vivo, as depicted in the figure. Essentially, in the in-vivo labelling approach, ferumoxytol was taken up by rat reticuloendothelial system and labelled BM-MSCs were obtained from the rat. The label was retained in the cells throughout the steps of harvesting and ex-vivo expansion. Compared to unlabelled control cells, in-vivo-labelled MSCs showed higher iron content and shorter T2 relaxation times upon transplantation into rats with osteochondral knee defects. In-vivo labelling approach is more effective, and it reduces the risks of contamination and biological alterations that can occur during the ex-vivo labelling procedures. However, in-vivo methods of labelling present limitations, as discussed in the review

Since MSCs exhibit poor ferumoxytol-uptake ability, earlier studies used protamine sulphate (clinically applicable, FDA-approved) [73] as a transfection agent to enhance ferumoxytol uptake. To overcome the issues of transfection-agent-induced undesirable effects and ferumoxytol’s incapability to effectively label MSCs in cell culture with or without protamine [74], subsequent studies established protocols that allowed ferumoxytol-labelling of MSCs without the need for transfection agents and/or electroporation. For example, MSCs were labelled with ferumoxytol in-vivo by intravenously injecting it in rats [75]. This interesting procedure is depicted and elaborated in Fig. 3.

Despite the advantages, the in-vivo method (Fig. 3) cannot be applied to autologous MSC transplantations because of the consequent inability to distinguish between the ubiquitously present labelled macrophages and transplanted MSCs. Also, since cell divisions can dilute ferumoxytol labelling to below MRI detection levels, the method would not be greatly suitable where MSC expansion is required to obtain sufficient MSCs for clinical application. As such, ex-vivo MSC expansion diminishes the MSC phagocytic ability [74]. Thus, another transfection-free approach was devised that utilised a “bio-mimicry” method for labelling MSCs with ferumoxytol. This approach mimicked the in-vivo environment of the MSCs. In this approach, MSCs retained their native features including cell size, recovered their phagocytic ability and showed ferumoxytol uptake that was comparable to that obtained by using transfection agents [74, 76]. This method has been successfully used in labelling MSCs with dextran-coated IONPs [77].

Ferumoxytol promises a wide range of clinical applicability. Osteoarthritis is a chronic joint inflammation characterised by cartilage degradation. Ferumoxytol-labelled MSCs helped determine that MSCs may modulate synovial inflammation. In a mouse model of osteoarthritis, intra-articular injection of ferumoxytol-labelled MSCs followed by MRI tracking showed sustenance of the signal in knee joints for up to 4 weeks. Also, there were decrements in MRI scores related to fluid oedema, pro-inflammatory cells and synovial fibrosis, and an increment in macrophage infiltration with preponderance of homeostatic macrophages, collectively demonstrating the anti-inflammatory characteristics of these labelled MSCs [78]. When ferumoxytol-labelled MSCs were transplanted in mice to alleviate calvarial defects, cells could be monitored and quantified over time via magnetic particle imaging, thereby promising useful MSC-based approaches for bone regeneration [79].

Alzheimer’s disease, a type of dementia, is characterised by constant decline of brain functions including thinking skills. Ferumoxytol-labelled human umbilical-cord-derived MSCs did not show cell toxicity, retained stemness in-vitro, and these cells were successfully engrafted into the brain of mice models of Alzheimer’s disease. Administrated MSCs could be detected in-vivo for up to 14 days at the site of injection via MRI. The success of this approach promised tracing of transplanted MSCs in real time in patients with Alzheimer’s disease [80].

In head and neck squamous cell carcinomas, smaller lymph nodes can harbour clinically occult metastases, which can make routine imaging modalities challenging. Previously, ferumoxytol was studied as an intra-tumoral contrast agent in MRI for intracranial malignancies and in prostate cancer. A subsequent study aimed to assess the feasibility of Ferumoxytol dynamic contrast enhanced-weighted MRI relative to gadolinium-based dynamic contrast enhanced-MRI for nodal and tumour imaging in five patients with head and neck squamous cell carcinomas. However, this study was prematurely terminated because of FDA black box warning [81].

### Dextran

Parkinson’s disease is a neurodegenerative disease characterised by the death of dopaminergic neurons. Brain iron accumulation is believed to play a role in the pathogenesis [82, 83]. Chung et al. exploited the migration and differentiation ability of MSCs and demonstrated for the first time that dextran-coated IONPs can enhance the therapeutic benefits of MSCs in a mouse model of Parkinson’s disease. IONP-dextran-labelled human BM-derived MSCs migrated towards the sites of damaged dopaminergic neurons in the mice brain. Also, these dextran-coated IONPs improved the ability of MSCs to rescue host dopaminergic neurons, increased MSC migration ability (due to elevated CXCR4, EGFR and IL-15R) (Fig. 2) and enhanced MSC differentiation into dopaminergic-neuron-like cells to replace the damaged neurons and secrete neurotrophic factors [5]. Similarly, MSCs appeared as a promising modality for Alzheimer’s disease when human Wharton’s jelly-derived MSCs were labelled with dextran-coated IONPs and magnetically delivered to the hippocampus of a rat model of Alzheimer’s disease. These rats showed memory and cognitive improvement [77].

Untreated chronic liver pathologies can progress through the stages of fibrosis, cirrhosis and cause predisposition to hepatocellular carcinoma [84]. Dextran-coated-IONP-labelled BM-MSCs for treatment showed promising results when these cells successfully engrafted in rats with fibrotic livers, and promoted fibrosis-regression and inhibited dysplasia [85]. This could be partly attributed to MSC’s anti-inflammatory effect as it can reduce the levels of macrophage-produced inflammatory cytokines like tumour necrosis factor (TNF)-α and IL-1β as well as TGF-β, and IL-6 (the latter two cytokines can be pro or anti-inflammatory). Also, MSCs demonstrate antifibrotic effects by secreting matrix metalloproteinases [86].

Interestingly, the efficacy of MSC therapy depends on the microenvironment of the injured liver. MSC transplantation into liver-damaged mice reduced liver fibrosis and the MSCs differentiated into hepatocyte-like cells and smooth muscle cells. However, in mice with liver fibrosis/cirrhosis but with macrophage depletion in the liver, fibrosis reduced significantly more than the former group without macrophage depletion. This difference was attributed to the pro-inflammatory and fibrogenic nature of macrophages in liver pathologies. Thus, data indicated that the hepatic niche can determine the outcome of the transplanted cells and therefore it should be altered for better clinical outcomes [86]. Along similar lines, administration of IONP-labelled BM-MSCs in rat models of cirrhosis and hepatocellular carcinoma helped restore normal histologic architecture, reduced cirrhosis, and reduced tumour mass, respectively. Here, IONP-labelled MSCs facilitated both detection and therapeutics simultaneously [87].

MSCs are known to exhibit both pro-tumour and anti-tumour effects [62]. IONP-dextran combination strengthened the anti-tumour effect of anti-tumour MSCs and reversed the pro-tumour effects of pro-tumour MSCs; thereby showing a potential application in MSC-based cancer therapy. In-vitro, dextran-coated-IONP-labelled human MSCs promoted more migration of the MSCs towards cancer cells compared to unlabelled human MSCs, partly due to IONP-dextran-induced elevation in CXCR4 (Fig. 2). Simultaneously, these IONPs inhibited colony formation of cancer cells (cell lines) and reduced the cancer-mediated angiogenesis and differentiation into fibroblasts of pro-tumour MSCs. Results were replicated in-vivo. When human MSCs labelled with dextran-coated IONPs were transplanted in a mice model of melanoma, an anti-tumour mechanism was activated in the MSCs, whereby pro-tumour mechanisms were inactivated that transformed pro-tumour MSCs into anti-tumour MSCs [51].

The chemokine C-C motif ligand-21 (CCL21) and the protein CD9 are amongst the many proteins and chemokines that play a role in cancer cell migration. Expression and secretion of CCL21 by human MSCs can facilitate chemotactic attraction to cancer cell lines. CD9 is a transmembrane protein that is expressed on several cell types including cancer cells and MSCs, and on the exosomes released from cells. It has a role in intercellular communications that mediate cancer cell migration and metastasis [43].

In a study, media of MSCs that were incubated with either ionomycin or dextran-coated IONPs were used to study migration of melanoma cell line. The study concluded that cellular CD9 had an inhibitory effect on CCL21 expression. Essentially, in human MSCs, ionomycin increased cellular CD9 expression, which reduced CCL21 secretion by MSCs, which lessened chemoattraction and thereby decreased the migration of melanoma cell line. In contrast, dextran-coated IONPs reduced MSC’s cellular expression of CD9, showed unaltered CCL21 secretion by MSCs (compared to control) and therefore unaltered chemoattraction and migration of melanoma cell line (Fig. 2) [43]. Thus, MSC treatment with dextran-coated IONPs and ionomycin helped envisage a pharmaceutical strategy involving CD9 to prevent cancer metastasis.

MRI tracking of injected IONP-labelled MSCs has shown promising results in animals but was not evaluated in humans until recently. Mathiasen et al. conducted the first contextual clinical study in human that evaluated the usage of dextran-coated TAT-conjugated IONP-labelled autologous BM-MSCs in intramyocardial transplantation of these cells in patients with chronic ischemic heart disease. Labelled MSCs could be tracked via MRI for up to 14 days after transplantation without compromising on safety [88].

Ferucarbotran (Resovist®) is an IONP coated with carboxydextran. Although it is still available in some countries like Japan, it has been discontinued from clinical usage since 2009 by several European countries [89]. Therefore, this IONP isn’t discussed here. Feridex, another dextran-coated IONP has been discontinued [89].

### Glucosamine

Glucosamine is a natural monosaccharide which makes the glycosaminoglycans found on several surfaces of joints (cartilage tissue). It is believed to provide chondroprotection and target prospective chondroprogenitors like the MSCs [90]. Its utility was further explored during IONP-labelling of MSCs. MSCs were labelled with IONPs, USIONPs and glucosamine-tagged USIONPs. Upon transfection with poly-lysine, the latter showed biocompatibility, better iron uptake by the MSCs and therefore higher MRI sensitivity in-vitro than the other combinations. Results replicated in-vivo where MRI was used for cerebral tracking of glucosamine-tagged USIONPs in rats. Data indicated that the presence of glucosamine (with poly-lysine) greatly enhanced cellular uptake of USIONPs and enabled better detection [91].

## IONPs in Combination with Non-carbohydrate Polymers for MSC Labelling

### Poly(L-Lysine): The Transfection Agent

There are a multitude of examples were poly-lysine has been used successfully used as a transfection agent, promoting IONP uptake by MSCs. Some examples are discussed here.

Photoacoustic imaging is a technique wherein following absorption of a short laser (light) pulse, the tissue expands due to heat and generates a photoacoustic wave. Essentially, the light pulse excites the contrast agent creating ultrasonic waves. The signal is detected by ultrasonic transducers and processed into a 3-D high-resolution image [27, 92]. This non-invasive imaging technique is accessible without ionizing radiation, offers deep penetration and higher resolution than MRI and enhances the contrast of traditional ultrasound at an affordable price. Prussian blue nanoparticles are currently being investigated as photoacoustic contrast agents [93]. Unlike the frequently used gold nanoparticles, Kim et al. labelled human MSCs with Prussian blue–poly(L-lysine) nanocomplexes for photoacoustic imaging. They prepared Prussian blue nanoparticles using a combination of ferric chloride and potassium ferrocyanide, complexed with poly-L-lysine, and labelled human MSCs with this complex. Cellular uptake of these particles was achieved, iron content was several folds higher than citrate-conjugated Prussian blue nanoparticles, and the cells could differentiate into adipocytes and osteocytes without alterations in viability, proliferation, and cytokine secretion. Labelled MSCs showed strong photoacoustic contrast in-vitro and in-vivo (in mice for 14 days), thereby offering good spatial and temporal resolution [93].

Later, Mishra et al. longitudinally tracked (for 21 days post-transplantation) IONP-poly-L-lysine labelled MSCs in mice with traumatic brain injury using MRI. Data revealed that damaged tissue was replaced with healthy tissue and MSCs incubated with the aforementioned complex in a ratio of 50:1.5 μg/ml for 6 h were optimised to increase the labelling efficiency [94]. Low molecular weight protamine is a non-toxic substitute of protamine with effective cell penetrating property [95]. When IONPs were conjugated with this non-toxic protein transduction domain, the iron content in the labelled MSCs was much higher than cells labelled with only IONP and IONP-poly-L-lysine. Also, the MSC differentiation capacity remained unaltered [96].

### Poly(Lactide-Co-Glycolide) and Poly(L-Lactide)

With time, there is a decrease in the MRI signal from the internalised IONPs because of cell proliferation and exocytosis of IONPs. Proliferation of IONP-labelled transplanted cells allows distribution of particles between the daughter cells leaving only a small percent of cells containing the particles after a few cycles. Xu et al. attempted to tackle this by exploiting two properties of nanoparticles: a) polystyrene-based microparticles exhibit a strong signal, and b) bigger particles show slower exocytosis rate. They hypothesised that 1–2 μm-sized particles with strong MRI signal would decrease particle exocytosis and enable better and longer longitudinal tracking of MSCs. Accordingly, biodegradable poly(lactide-co-glycolide) microparticles loaded with IONPs were used to label MSCs. In-vitro and in-vivo experiments (in mice) revealed enhanced cellular localisation of iron, which increased the signal to noise ratio and offered better detection via MRI. Larger particle size increased resident time within the cells and provided longer detectable time of labelled MSCs in comparison to IONPs alone. This occurred without compromising MSC viability, proliferation, migration and homing at the sites of inflammation [97].

In another study, IONPs were encapsulated in poly(lactide-co-glycolide) microspheres and those with 1.38% IONPs showed highest adherence of BM-MSCs in-vitro after 48 h of co-culture. These microspheres significantly promoted osteoblast differentiation of BM-MSCs, as evidenced by detecting alkaline phosphatase (ALP), collagen-1, osteopontin and osteocalcin; the osteogenesis-related proteins (Fig. 2). Administration of these microspheres into rat femoral bone together with an external magnetic field remarkably improved the bone defect [98].

Preservation of MSC functionality is a prerequisite for the success of MSC therapy. Human MSCs were successfully labelled with iron oxide-poly(L-lactide) nanoparticles and the fluorescent dye *N*-(2,6-diisopropylphenyl)-perylene-3,4-dicarbonacid-imide without any impact on viability, differentiation, proliferation, clonogenicity, adhesion, and other immunomodulatory and phenotypic properties. Following subcutaneous implantation in rats, these could be detected via MRI for several weeks [99].

### Polydopamine

Dopamine is a natural neurotransmitter that can spontaneously form polydopamine via autopolymerisation in-situ [100]. Polydopamine is biodegradable, biocompatible and therefore, widely used to coat IONPs for biological applications [2]. Polydopamine-capped IONPs were successfully incorporated in MSCs without alteration in MSC stemness. This labelling enhanced their migration ability via CXCR4 upregulation, as demonstrated via accumulation of labelled MSCs at burn injury sites in a rat model. Labelled cells also exhibited healing properties (increased anti-inflammatory factor IL-4), as indicated by reduction in inflammation at the injury sites (Fig. 2), thereby showing the utility of this approach in healing burn wounds [57].

In rat BM-MSCs labelled with polydopamine-capped IONPs, the latter improved the biocompatibility and stability of the IONP core. In-vitro, polydopamine-capped IONPs retained MSC differentiation characteristics. However, there were no major differences in results between MSCs labelled with polydopamine-capped IONPs and.

MSCs labelled with polydopamine-devoid IONPs in the following parameters: IONP-induced reduction in inflammation (in-vivo), MSC release of VEGF (which promotes angiogenesis and neovascularisation of damaged tissue) (in-vitro) and migration (in-vitro). Interestingly, MSC expressions of migration-related proteins c-Met and CCR1 induced by IONPs alone (Fig. 2) far exceeded the levels induced by polydopamine-capped IONPs [2]. Thus, polydopamine-capping of IONP can offer advantages but its usage should consider the outcome desired.

Similarly, human umbilical cord MSCs were labelled with polydopamine-coated IONPs. In-vitro, labelled MSCs showed hemocompatibility, biocompatibility, low cytotoxicity and enhanced migration ability. Labelled cells were then administered in the femoral head in rat models of osteonecrosis, a chronic irreversible disease in human that eventually leads to joint dysfunction. In presence of an external magnetic field, these cells enhanced osteogenic effects, as evidenced by elevations in Runx-2 and Osterix (osteogenic proteins) and Akt and Bcl-2 (anti-apoptotic proteins), and decrements in caspase-3 and Bad (apoptotic proteins) (Fig. 2). Compared to controls, in cases of polydopamine-labelled MSCs, rat serum levels of IL-4 and IL-10 (anti-inflammatory cytokines) were higher and TNF-α and IL-6 (pro-inflammatory cytokines) were lower. The effects were even more pronounced in presence of the external magnetic field. These collectively mediated bone repair and suggested a novel strategy for treating osteonecrosis [100].

## IONPs in Combination with Elements for Labelling MSCs

### Ruthenium/Selenium

Human umbilical cord MSCs were labelled with IONPs bound to ruthenium (γ-Fe_2_O_3_@Ru) and selenium (γ-Fe_2_O_3_@se). Treatments elevated gene expressions of bone morphogenetic protein (BMP), ALP, RUNX-2, osteocalcin, osteopontin, collagen-1α and p-SMAD-1/5 in MSCs (Fig. 2) [101]. This reminds us of the iron-induced upregulation of the TGF-β pathway components TGF-β receptor-2 and p-Smad-2 observed in murine hepatic stellate cells [102]. Thus, the aforementioned elevation in p-SMAD-1/5 in MSCs could be due to the iron component in γ-Fe_2_O_3_@Ru and γ-Fe_2_O_3_@se. MSC differentiation into osteoblasts was induced without cell cycle arrest or oxidative stress. However, Fe_2_O_3_@Ru diffused more effectively in the cytoplasm, localised in the nuclei of MSCs, strongly induced osteogenesis and inhibited adipogenic differentiation [101]. Indeed, where MSCs were cultured with Fe_2_O_3_@Ru, p-SMAD-1/5 was further upregulated and peroxisome proliferator-activated receptor gamma (PPARγ) (adipogenic marker) was downregulated (Fig. 2). This indicated that Fe_2_O_3_@Ru-mediated preferential osteogenic differentiation of MSCs could be regulated by the BMP-SMAD-1/5 pathway [101], which usually modulates the upregulation of osteogenic genes and down-regulation of adipogenic genes [3, 101].

### Gold

IONPs were combined with gold so that the nanoparticle could be detected by both MRI and photoacoustic imaging; the latter offers several advantages, as discussed previously. Essentially, GFP-conjugated BM-MSCs were successfully labelled with gold-coated IONPs, which did not alter MSC migration and differentiation capacities in-vitro. Labelled MSCs were introduced in mice through the carotid artery to visualise these cells in the brain tumour of mice and assess MSC homing via MR and photoacoustic imaging. After 72 h of injection, the photoacoustic signal emitted from the tumours of labelled MSCs was more enhanced than tumours with unlabelled MSCs. MRI matched the results of photoacoustic imaging and showed significant progressive (over time) hypointensity of the tumour with gold-coated IONP-labelled MSCs. Histological examination revealed co-localisation of GFP fluorescence and iron, which indicated that gold-coated IONP-labelled MSCs carried the IONPs even after 72 h of injection. Notably, during initial in-vitro experiments, lipofectamine was used as a transfection agent which enhanced the uptake of gold-coated IONPs and did not affect viability but decreased proliferation. Lipofectamine was not used in subsequent in-vivo experiments because it further decreased proliferation of labelled MSCs and reduced MSC viability over time [92], thereby providing an example of the disadvantage of using transfection agents.

Despite the therapeutic effects of MSCs, these can show low viability, poor homing and a potential risk of oncogenesis induced by direct MSC administration at the wound site. Im et al. aimed to mitigate these limitations by exploiting the ability of endocytosed IONPs inside the endosomes to undergo degradation into ions due to the low pH of the endosomes. Thus, they designed pH sensitive nanoparticles that allowed endocytic release of iron ion inside the MSCs. High-passage-number (P12) MSCs were treated with gold-iron nanoparticles. Intracellular iron ions elevated MSC HIF-1α expression, which in turn elevated VEGF expression (essential for wound healing). This resultant iron-ion-induced upregulation of VEGF expression in these high-passage-number labelled MSCs was comparable to the amount released by low-passage-number MSCs without the gold-iron nanoparticle treatment. Administration of this high-passage-number-derived labelled conditioned medium into a mouse model of skin wound led to better angiogenesis compared with the group injected with unlabelled conditioned medium from high passage MSCs. Results obtained were similar to that in mice injected with conditioned medium from low passage MSCs; the results were enhanced angiogenesis, re-epithelization and tissue remodelling [103]. This study introduced a new approach of using MSC conditioned medium instead of MSCs to mediate MSC therapeutics.

### Zinc

Zinc ions are generally redox stable and induce antioxidant effects in cells [104]. Like others, Kim et al. used the principle of stimulating gene expression via intracellular ions and hypothesised that the combination of two metal ions, zinc and iron (dual ion delivery system) will induce zinc-ion-mediated osteogenesis and iron-ion-mediated paracrine secretion by the MSCs. Thus, aiming to improve the osteogenic differentiation of human MSCs and promote angiogenesis simultaneously, zinc-based IONPs were synthesised. These were dissolved in a weak acid that mimicked low pH within the endosomes. In-vitro, zinc-IONP-labelled MSCs showed enhanced osteogenic expression (RUNX-2 and ALP) and increased secretion of VEGF (angiogenic paracrine factor) (Fig. 2). The concurrent intracellular release of zinc and iron ions modulated the subtle increase in ROS in the MSCs without causing cytotoxicity. This augmented MSC osteogenic potential and promised useful applications of this approach in future [104]. Notably, if MSC therapeutic efficacy is to be enhanced via delivery of intracellular ions, then it is crucial to adjust the concentrations of the ions delivered. This is because intracellular ion homeostasis is essential for cell viability and functionality. For example, high zinc ion concentration can dysregulate intracellular iron ion homeostasis, cause cellular iron overload and induce iron-ion-mediated cell death called ferroptosis [105].

To tackle the challenge of compromised stem cell labelling efficiency and low stem cell homing efficiency, MSCs were labelled with zinc-doped IONPs in combination with the amphiphilic polymer hyaluronic acid–cholanic acid. This allowed effective MRI-guided delivery of MSCs to the target sites, helped track MSCs in-vivo and improved MSC homing into mice models of glioblastoma and traumatic brain injury. This occurred without notable cytotoxicity and phenotypic alterations in MSCs, thus allowing targeted delivery of stem cells [56].

## IONPs in Combination with Compounds and/or Stains for MSC Labelling

### Silica

MSC-labelling with IONPs in combination with silica (and associated elemental complexes) has gained foothold in the field. When human MSCs were labelled with silica-coated IONPs along with FITC-incorporated mesoporous silica, IONP uptake was enhanced without affecting cell viability and differentiation ability [106]. Distinct from several previous studies that used transfection agents to promote IONP uptake by MSCs, Harrison et al. showed that silica-coating of IONPs to label human MSCs allowed rapid cellular uptake of the particles without any transfection agent. MSC viability, surface markers and differentiation capacity remained unaltered. When silica-IONP-labelled MSCs were seeded on a porous collagen scaffold, followed by exposure to a magnetic field, these cells showed directed migration in-vitro. This could be very useful in seeding procedures for 2D culture and 3D tissue-engineered scaffolds and can reduce the cell dosage required for Prochymal therapy [35].

Additional surface modification of silica-coated IONPs with amine not only allowed incorporation into BM-MSCs and localisation in lysosomes in the absence of transfection agent, but also increased the labelling efficiency and MRI detection sensitivity by 2–5 fold [107]. Similarly, BM-MSCs labelled with amine-modified-silica-coated IONPs retained cell viability, osteogenic and adipogenic differentiation potential, and increased migration ability in-vitro (increased SDF-1 and CXCR4 expressions) (Fig. 2). When injected in rabbits with osteonecrosis bone defect, the label helped to track MSC homing at the defect site via MRI; homing being accelerated via poly(lactic-co-glycolic acid)/tricalcium phosphate scaffold to mediate tissue repair [108]. This showed the potential of this system not only in tracking MSCs but also in accelerating homing of the transplanted MSCs onto injury sites.

A concern in labelling MSCs with IONPs is oxidative damage to DNA, lipids and proteins. Novotna et al. observed oxidative damage in human BM-MSCs due to IONP-labelling, so lowering the IONP concentration was recommended [109]. Following this, labelling of rat MSCs with a non-toxic dose of silica-coated cobalt-zinc-iron nanoparticles (using poly-L-lysine) did not show oxidative damage to macromolecules. Subsequent transplantation of labelled rat MSCs into rat brain showed no increase in oxidative damage to the macromolecules in the brain, compared to control unlabelled MSCs. Therefore, this method was considered safe for tracking transplanted cells via MRI [110]. Thus, silica-coated IONPs showed a huge potential in targeted cell-based therapy and in mediating theranostics, which is a combination of therapy and diagnostics.

### Polyethylene Glycol

A potential for theranostics was also observed when placenta-derived MSCs were used to ameliorate mouse glioblastoma, an aggressive type of brain and spinal cord cancer in human. Hsu et al. labelled these MSCs with polyethylene-glycol-coated IONPs. Polyethylene glycol coating to IONPs allowed better uptake of these particles by MSCs and showed no cytotoxicity or alteration to MSC proliferation or stemness. Labelled cells were injected in mice and tracked for movement towards the tumour cells (glioblastoma stem-like cells that are thought to be responsible for glioblastoma tumour instigation and sustenance) in real time via MRI. Mediated via the chemokine ligand 5 and augmented by preconditioning with hypoxia, these placenta-derived MSCs showed better migratory potential towards glioblastoma stem-like cells and across the blood-brain barrier compared to BM-MSCs. The success of this approach promised tracing of the infiltration of therapeutic MSCs into the tumour, and helped envisage targeted therapy for brain tumours in the future [111]. Likewise, IONP-labelled human amniotic membrane-derived MSCs exerted protective effects against isoproterenol-induced myocardial injury in rats by improving cardiac function and reducing fibrosis. This involved reducing inflammation via the NF-κB/MAPK pathway; injury induced elevation in NF-κB was reversed by labelled MSCs in presence of an external magnet [112].

### Combination of Silica and Polyethylene Glycol

While a complex of iron-iron oxide (Fe@FeOx) nanoparticles has been recognised as a promising MRI contrast agent for long, silica-coated Fe@FeOx nanoparticles accompanied by polyethylene glycol chains improved their biocompatibility and showed better darkening effect than Resovist® (Ferucarbotran), the reference contrast agent available in Japan. This combination neither showed any major cytotoxicity on human colon cancer cell line and non-tumoral fibroblast cell lines nor haemolytic activity against human RBCs, thus proving as a promising combination to detect early small tumours [113].

### Fluorophore

Considering the potential of human MSCs to promote repair after stroke, clinical-grade human MSCs were labelled with fluorophore-tagged micrometre-sized IONPs without the use of transfection agent. Data showed no alterations in MSC viability, phenotype, or differentiation capacity in-vitro. When these labelled MSCs were introduced in a rat model of stroke, MSCs could be tracked in-vivo via MRI and fluorescent microscopy for up to one month after MSC administration. This approach can be very useful to formulate optimal route and dosing of cells, particularly for in-vivo MRI studies following stroke. However, it raises the usual concern of the release of IONPs from dead MSCs [114].

### Rhodamine B

Pavon et al., by using the IONP-rhodamine B combination, showed that the MSCs can contribute to tumour progression. CD133+ stem cells of glioblastoma (brain/spinal cord cancer) secrete various chemokines including MCP-1 and SDF-1 that play a role in MSC tropism. Cultured human umbilical cord blood MSCs expressed the receptors for these secreted cytokines, namely CCR2 and CXCR4, respectively, and their associations induced MSC migration towards the CD133+ stem cells of glioblastoma (Fig. 2). When IONP-rhodamine-B-labelled MSCs were administered into the caudal vein of rats, these MSCs crossed the blood brain barrier and showed migration towards the tumour. However, later, this led to tumour development and invasiveness, and showed a large number of cycling cells, indicating promotion of tumour growth, an undesirable event. Therefore, the usage of MSCs as carriers of therapeutic materials to tackle cancer should be applied with great caution [50].

In the absence of transfection agent, BM-MSCs were labelled with silica-coated fluorescent magnetic nanoparticle (ferrite core) containing rhodamine B isothiocyanate. When these were intra-splenically injected, cells could be successfully tracked in a rat model of liver cirrhosis, providing both optical and magnetic features [115]. Similarly, IONP-rhodamine B (and oleic acid) combination was used to label mouse BM-MSCs. In-vitro, this induced the expression of ROS and CXCR4 in the MSCs, and together with the elevated expression of SDF-1 in the injured tissue, this likely activated CXCR4-SDF-1 signalling; a mechanism known to enhance chemotactic activity (Fig. 2). Following the intranasal delivery of labelled MSCs in mice with olfactory bulb injury, MSCs were guided by a permanent magnet to promote migration to the injured olfactory tissue so that homing of MSCs could be enhanced in-vivo [55].

Despite successful cell homing in-vivo, whether the usage of stains negatively affected cell properties required confirmation. Thus, different vital stains were assessed in-vitro. MSCs labelled with Molday ION Rhodamine B™ (rhodamine B labelled-IONP) retained MSC viability and differentiation potential, increased sensitivity [116] and showed some superior results compared to MSCs labelled with CellTracker™, Green CMFDA, and eGFP-mRNA (genetic pre-tag). Labelling MSCs with the latter group of stains altered the metabolic activity and morphology of the MSCs. Overall, while the gene expression of the growth factors *CNTF, BDNF*, *NT3* and *PSAP* remained stable post labelling, alterations were observed in the gene expressions of the growth factors *IGF*, *EGF*, *GDNF* and *HGF* [117]**.** Thus, further experiments are necessary to rigorously assess the effects of stain-tagging to MSCs and evaluate whether this approach has essential and/or superior clinical benefits.

### DAPI

An issue with MRI tracking in-vivo is the detection of signal generated by macrophages that engulfed IONPs released from dead cells. 4′, 6-diamidino-2-phenylindole dihydrochloride (DAPI), a fluorescent DNA dye, is frequently used to label and trace stem cells as well as visualise nuclei under fluorescent microscope. Therefore, labelling MSCs with DAPI in addition to IONPs can resolve the aforementioned issue. Rat BM-MSCs were co-labelled with Feridex (withdrawn from market) and DAPI using poly-L-lysine as transfection agent. When labelled cells were transplanted via hepatic artery into rat livers, cells could be detected in-vivo via MRI for up to 7 days post transplantation [118]. In another study, rabbit MSCs were co-labelled with IONPs and DAPI and transplanted via intravenous injection into rabbits with liver tumours to assess MSC tumour-homing capacity via MRI. Histological analysis revealed that IONP distribution, as visualised via Prussian blue stain, matched with the DAPI stained fluorescent nuclei i.e. iron co-localised with DAPI. These cells localised in the tumour with high specificity and could be tracked for 7 days in-vivo via MRI [49].

### Prussian Blue Nanocubes

The inability to track the transplanted cells in real time hinders the full potential of cell therapy and regenerative medicine, particularly in spinal cord injuries. To tackle this, specifically formulated Prussian blue nanocubes (PBNCs) that contain IONPs were prepared to achieve multimodal MR and photoacoustic contrast. Human adipose-derived MSCs were labelled with PBNCs and labelled MSCs were injected into rat spinal cord ex-vivo while ultrasound and photoacoustic images were acquired. These imaging approaches guided the needle during MSC injection, and MRI imaging was enhanced as PBNCs acted as contract agents. Thus, a combination of ultrasound, photoacoustic imaging and MRI was used to allow multimodal detection. This approach detected low concentration of MSCs and tracked cells in the spinal cord post-administration, thereby promising applications in intra-operative and post-operative situations [119]. Similar PBNCs have been designed by other groups that can act as photoacoustic and MR contrast agents and are useful for diagnostic and therapeutic applications [120].

## IONPs in Combination with DNA Improve MSC Transfection

**Fig. 4:**
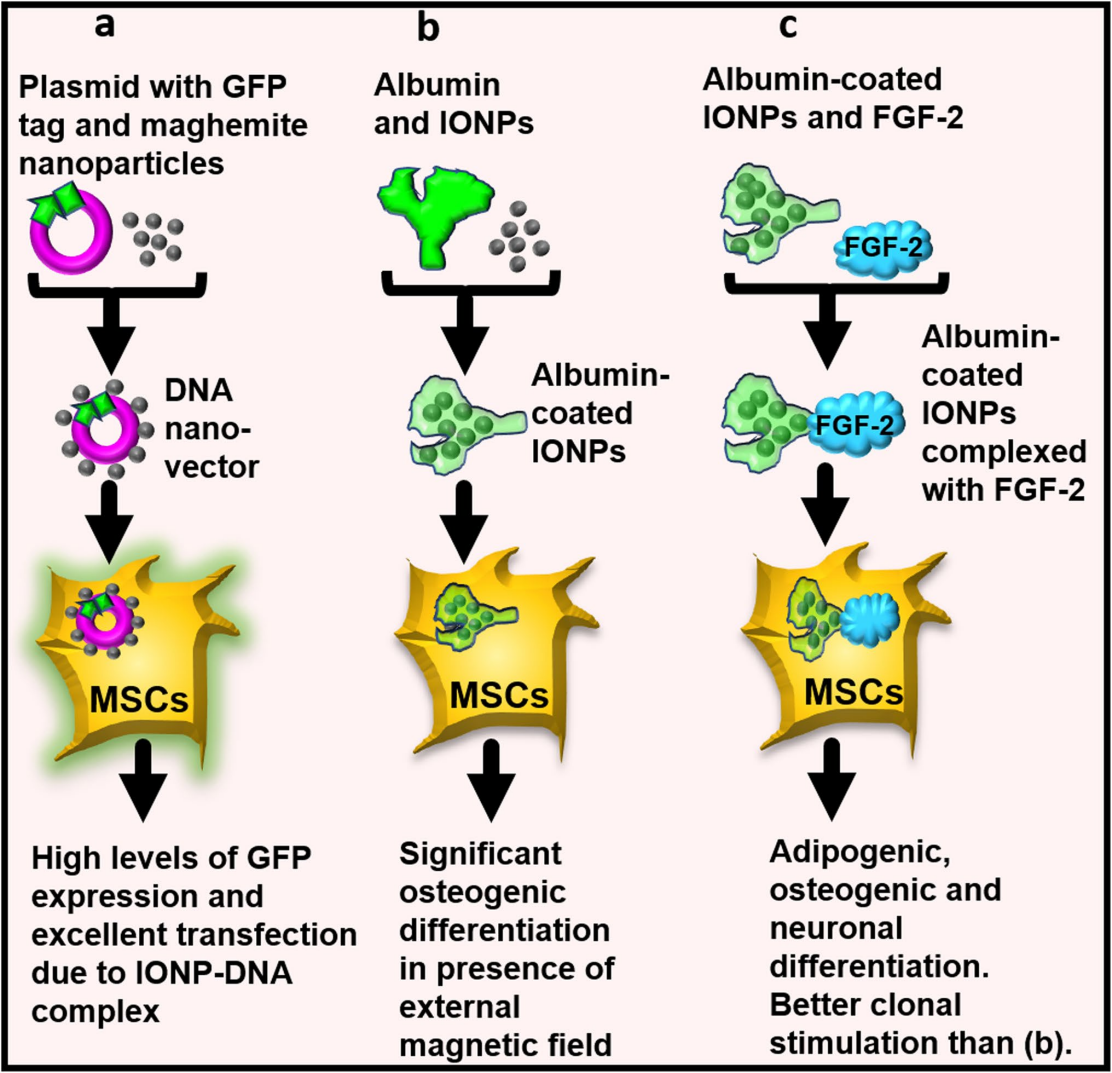
Principle of labelling MSCs with iron oxide nanoparticles (IONPs) bound to DNA, albumin, and Fibroblast growth factor-2 (FGF-2). Schematic showing (**a**) IONPs bound to plasmid DNA, (**b**) IONPs bound to albumin and (**c**) albumin-coated IONPs bound to FGF-2. Consequences of endocytosis of these complexes by the MSCs have been stated

Due to the limitations of viral vectors as gene delivery systems, a non-viral transfection approach like usage of DNA vectors is sought for biomedical applications. Distinct from the commonly used coated IONPs, Margo et al. used naked surface active maghemite nanoparticles. These uncoated IONPs were bound directly and covalently to DNA, and these acted as vectors for MSC transfection. Essentially, a plasmid containing a GFP tag (gene) was directly absorbed onto surface active maghemite nanoparticles, which formed a DNA nano-vector. This was spontaneously internalised by horse-blood-derived MSCs, and the cells showed higher levels of GFP than that expressed in cells transfected by lipofectamine, even without an external magnetic field (Fig. 4a). Excellent transfection efficiency was observed, which was attributed to the physical/chemical and hydrodynamic properties of the IONP-DNA complex. In essence, the method demonstrated a reliable tool for gene delivery and emergence of a novel DNA nano-vector that can help achieve effective MSC transfection [121].

## IONPs in Combination with Albumin and Fibroblast Growth Factor-2 (FGF-2)

The most abundant serum protein albumin is widely used in research because of its biodegradable nature, high stability in blood, and biocompatibility [122]. Coating IONPs with albumin can prevent particle agglomeration, and albumin is almost inert for differentiation of cells. Albumin-protected IONPs were internalised by BM-MSCs and these stimulated significant MSC osteogenic differentiation under a constant static magnetic field (Fig. 4b). This was indicated by increments in ALP activity and calcium deposition, along with elevations in the mRNA and protein expressions of osteocalcin and collagen I (Fig. 2) [123]. The complex of albumin and IONPs can bind to various macromolecules with high affinity. Levey et al. exploited this property. They conjugated albumin-coated IONPs to FGF-2 and observed the effect of this complex on human BM-MSCs in-vitro. Data showed that following endocytosis, the presence of FGF-2 enhanced MSC proliferation and promoted differentiation into adipogenic, osteogenic and neuronal lineages (Fig. 4c). Conjugated FGF-2 was able to better stimulate MSC clonal expansion than free growth factor, likely because it was stabilised against the action of enzymes and inhibitors in the serum and tissue culture [40]. These results help envisage wide biomedical applications of such complexes and better in-vivo detection of MSCs via MRI.

## IONPs Complexed with Drug

Human triple negative breast cancer is the most aggressive type of breast cancer. Doxorubicin is one of the recommended drugs to treat breast cancer. In dealing with cancer, there are issues such as drug-induced resistance, therapeutic inefficacy to tumours, systemic toxicity and lack of a system that allows regulated drug release to tumorous tissues. To overcome some of these issues, complexes including a combination of IONPs and dugs have been designed. A combination of lipids, doxorubicin (drug), gold nanorods and IONPs form a complex called LDGI. MSCs were successfully labelled with LDGI for photoacoustic imaging, targeted photothermal therapy and chemotherapy in mice models of breast cancer, aiming to apply this targeted therapy to human breast cancer. In-vitro, the label was successfully taken up by the MSCs and did not alter cellular functions. Labelled MSCs maintained tumour tropism and the IONPs in the LDGI complex were found to upregulate CXCR4 on the MSCs (Fig. 2) [124]. In vivo, the complex provided a means of releasing the drug doxorubicin to promote cell apoptosis (via chemotherapy) and allow photothermal therapy simultaneously upon light irradiation. The MSC-LDGI complex was the most efficient in inhibiting tumour growth [124], which promised a clinically translatable drug delivery system via the MSCs.

Cell-membrane-coated nanoparticles show diagnostic and therapeutic applications. Recently, doxorubicin-loaded IONPs were coated with MSC membranes, and the therapeutic efficacy of this complex in treating colon cancer was examined in-vitro and in mice models. The complex showed better uptake by tumour cells, anti-tumour properties and reduced side-effects compared to the complex without MSC membranes [125]. This promised an excellent strategy for targeting tumours with specific systems that can deliver therapeutic drugs.

## Specific Caveats Associated with IONP-Labelling of MSCs

Difficulties related to MSC labelling with IONPs and MSC detection, and possible solutions have been tabulated in Table 3. The list is not exhaustive.

**Table 3:** Challenges and putative solutions related to IONP-labelling and subsequent detection of MSCs

Issue	Details	Putative solutions
Weak phagocytosis, low iron content & low sensitivity	MSCs are phagocytic but they become less phagocytic over time during ex-vivo culturing [74, 75]. As such, stem cells are generally less able to uptake IONPs than macrophages [33]. Thus, IONP-labelling of MSCs demonstrates low efficiency, low iron content per particle, reduced uptake in MSCs and weak MRI signal [97].	• To assist incorporation into cells, IONPs could be cross-linked with a signal peptide/cell penetrating peptide that facilitate membrane translocation (e.g. HIV1- Tat protein) [36, 88]. • Transfection agents (protamine sulphate, lipofectamine & poly-lysine) have been used to facilitate IONP entry into MSCs and enhance cell/tissue visualisation by MRI. These bind to IONPs via electrostatic interactions rather than chemical conjugation [36].
Transfection agent-induced cytotoxicity	Transfection agents may alter cell biology leading to undesirable side-effects/cell toxicity [126].	• Transfection-agent-free IONP-uptake can be achieved [35, 74, 76, 114, 127, 128]. • IONPs can be coated with a polymer to rescue cell viability [129].
Alteration in MSC properties	IONPs with or without transfection agent can reduce colony formation ability and migration capacity of human MSCs; the effects being abolished after 2 passages [130]. IONPs with or without surface coating can alter gene expression, viability and differentiation potential [108].	In a study, treatment of MSCs with a combination of IONPs and hypoxia exerted synergistic effects. It improved MSC migration and chondrogenic differentiation in-vitro and demonstrated corresponding remedial effects in-vivo [131].
Poor uniformity of IONPs	IONPs have been proposed to aid in gene delivery but this is challenged by poor uniformity of IONPs that hamper the magnetic property and reproducibility, leading to ineffective gene delivery.	Xu et al. developed uniform 15 nm-sized IONPs that in the presence of a magnetic force showed the potential for an efficient gene delivery into human MSCs without negatively affecting their proliferation and multilineage differentiation [132].
Decrement in cellular IONP content with cell proliferation	The cellular content of IONPs decrease with cell proliferation in-vivo, which can diminish detection.	MSCs can be genetically modified and transfected with one or more genes from the magnetotactic bacterium *Magnetospirillum magneticum AMB-1.* This imparts MSCs with an ability to accrue more iron because specific genes from this bacterium can help synthesise magnetic crystals that are incorporated into magnetosomes [71].
Inaccessible sites and low MSC retention in-vivo	In some clinical cases, lesions may be in sites that are difficult to access such as joints, spinal cord, and heart. Low retention of MSCs at these sites can reduce the efficacy of cell therapy.	• Static magnetic fields can be used to guide the IONP-labelled MSCs to the sites of injury in-vivo and achieve focussed and long lasting retention [133]. • Also, placing an external magnet near the organ of interest, for example liver, can attract the IONP-labelled MSCs and fasten cell homing into the liver [33].
Issues in using static magnetic field	Usage of static magnetic field can alter MSC properties such as viability, proliferation, differentiation and extracellular vesicle secretion [2].	IONP-labelling has shown to promote MSC migration to injury sites in-vivo in the absence of an external magnetic field. This avoided the possibility of MSC alterations due to static magnetic fields and promised an effective MSC-based therapeutic strategy [2].
IONP elimination from the system	In-vivo, IONPs are recognised as foreign bodies, so these are rapidly removed by the reticuloendothelial system [112]. While this is an advantage, it can also prevent detection.	IONPs can be conjugated with polymers like polyethylene glycol that improve their stability and reduce toxicity [112].

In addition to the caveats discussed so far, IONP-labelling of MSCs poses challenges like decrement in cellular iron content upon cell proliferation, which leads to progressive loss of detection signal, expulsion of IONPs from labelled cells, engulfment of transplanted cells by macrophages resulting in false positives and an inability to detect cell differentiation [27, 36]. In-vitro, cell division can cause the dilution effect and lead to progressive decline in labelling with increasing culture time [35]. For example, citrate-coated-IONP-labelled adipose-derived stem cells retained their ability of multilineage differentiation in-vitro, but the percent of labelled cells decreased during the 4-week in-vitro expansion [38]. Collectively, this makes long-term tracing difficult and may not reflect the true number of transplanted cells.

IONP concentration can determine MSC proliferation and differentiation ability. For example, although IONP-labelled umbilical cord MSCs could be successfully tracked in rat brains, labelling these MSCs with 100 μg/mL IONP decreased proliferation and influenced their differentiation ability into cartilage in-vitro [134]. Moreover, IONP uptake by MSCs is IONP-size dependent. For example, human foetal MSCs labelled with 600-nm microgel iron oxide particles (amongst a range sizes) showed 3–6-fold higher iron-loading than Ferucarbotran without affecting MSC proliferation or tri-lineage differentiation. In a rat photothrombotic stroke model, labelled MSCs successfully migrated from contralateral cortex to the site of stroke injury and showed 5–7 times higher MRI sensitivity than detection by Ferucarbotran. Also, it showed low cell toxicity, thus making this approach useful for tracking MSCs using clinical MRI scanners. However, due to necrosis and inflammation in the rat models, the transplanted human MSCs could not survive beyond 12 days [135]. Determination of long-term effects is pending.

IONPs are composed of iron, so these may generate ROS due to IONP-derived iron feeding into Fenton reaction. Due to their perinuclear localisation, IONPs can get access to cell transcription components and may alter gene expression directly. Also, IONP-entry-induced ROS may alter cell signalling pathways. While some of these changes can be beneficial (Fig. 2), these could also lead to toxic effects including morphological modifications, activation of immune responses and upregulation of redox-sensitive transcription factors [27]. Researchers have addressed some of these issues concerning IONP-labelling of MSCs and have proposed ways of tackling these challenges (Table 3).

## Impact of Similarity/Dissimilarly between MSCs from Different Sources on IONP-Labelling and its Outcomes

MSCs can be sourced from various body tissues. It is important to address whether there is biological similarity between MSCs obtained from these different sources. All MSCs show similar phenotypic properties and possess fibroblast-like morphology. However, there are some differences. For example, although adipose-tissue-derived MSCs and BM-MSCs share several biological features, there are some minor differences in their transcriptomes and proteomes (as expected), immunomodulatory activities and differentiation potential [136]. Contextually, BM-MSCs show better osteogenic and chondrogenic potential than adipose-tissue-derived MSCs. The latter cells demonstrate better adipogenic capacity [137, 138] and better retention of trilineage differentiation capacity over time than BM-MSCs [139]. Note that these cell populations are heterogenous and therefore do not constitute a single cell type [3]. This may be an additional reason for the subtle differences. While both are suitable for clinical applications, MSCs from one source might be more suitable than MSCs from another source for a particular clinical application. For example, despite the ability of adipose-tissue-derived MSCs to regenerate bone, BM-MSCs would be more suitable for applications related to bone regeneration because of their higher potential to serve the purpose [140].

There are other examples that show some similarities and differences in MSCs from different sources. For example, MSCs from dental-pulp and umbilical cord have shown higher proliferation than BM-MSCs and adipose-tissue derived MSCs. In the context of pluripotency, dental-pulp MSCs have shown slightly different gene expression profile (relative gene expression) from the MSCs of bone marrow, adipose tissue, and umbilical cord, while the latter three MSCs have shown similarity in this aspect. Thus, dental-pulp MSCs showcase their neural crest origin and differ slightly from the MSCs of these three sources [141]. BM-MSCs and neural stem cells are similar in their migration capacity and tropism for brain tumours [142, 143]. Also, both BM-MSCs and adipose-tissue-derived MSCs show pericyte-like characteristics and show similar tumour migration abilities. Thus, either could be used for targeting glioma [144]. Dental-pulp MSCs showed a more robust ability for osteogenic differentiation than adipose-tissue-derived MSCs [67]. Thus, the desired clinical outcome can better guide the choice/source of MSCs to be used.

Yet another interesting question is whether iron labelling works equally well for MSCs derived from different sources. This depends on the parameter being assessed and the MSCs being compared. For instance, in an in-vitro study, both adipose-tissue-derived MSCs and BM-MSCs could be IONP-labelled and then tracked with equal ease. Their responses to IONP-labelling were similar; both showed decrements in differentiation potential (adipogenic and osteogenic) following IONP-labelling, an increment in iron content, and a decrement in cell viability with increasing concentration of IONPs [145]**.** Another in-vitro study compared IONP-labelled MSCs from adipose tissue and Wharton’s jelly. Here, IONP-labelled adipose-tissue-derived MSCs showed better proliferation and lower senescence than unlabelled counterparts and Wharton’s jelly MSCs. IONP-labelled Wharton’s jelly MSCs showed reduced viability in comparison to unlabelled Wharton’s jelly MSCs. But this was not the case with adipose-tissue-derived MSCs. Also, IONP-labelling and magnetic field exposure promoted chondrogenesis in human adipose-tissue-derived MSCs but not in Wharton jelly MSCs [146]. Essentially, the chondrogenic potential of MSCs obtained from one source could be enhanced with magnetic stimulation but this did not occur in the MSCs from another source. Based on this data, adipose-tissue-derived MSCs would be more suitable than Wharton’s jelly MSCs for cartilage engineering. This clearly indicates that the source of MSCs determines its potential therapeutic usage, and IONP-labelling may (or may not) have differential effects on MSCs from different sources; conclusion on the latter will depend on the parameter being examined.

## Summary

Prior to administration, MSCs are often labelled with contrast agents to facilitate post-transplantation detection via MRI. MSCs can be labelled with IONPs that act as good MRI contrast agents that improve the detection and tracking of administered MSCs and in some cases enhance MSC therapeutic potential and augment regenerative medicine. For labelling, IONPs can be complexed with carbohydrates, non-carbohydrate polymers, elements, compounds, stains, drugs, growth factors and DNA; the latter in combination with IONPs acts as a DNA nano-vector. The advantages, limitations, and applicability of these approaches, as discussed here, need to be considered appropriately before embarking on further MSC-related clinical trials.

## Current State, Hurdles, Future Work, and Perspectives

There are several human studies wherein MSC therapy has shown success. For example, in clinical trials on graft-versus-host-disease [147–149], osteoarthritis [150–152] multiple sclerosis [153], pulmonary diseases [154–156] and inflammatory bowel disease [157, 158]. When Wharton’s jelly MSCs were used to ameliorate acute myocardial infarction, gadolinium-enhanced cardiac MRI, echocardiography, and ECG-gated single photon emission computed tomography were used. The approach was found to be safe and effective [159]. In all these studies, the focus was on directly examining the primary and secondary clinical outcomes post transplantation rather than detecting the location, survival, and differentiation of MSCs at the target site and then relating this to the clinical outcome. Indeed, MRI was used in some studies, but this was to examine alteration, regression, or amelioration of the condition/tissue following treatment. Success achieved in these studies (in terms of achieving the desired clinical outcomes) implied MSC-treatment efficacy and appropriate MSC functionality at the target site.

The aim of cell therapy is to regenerate/rejuvenate the damaged tissue so that the original tissue function is restored without causing cytotoxicity or inducing any aberrant processes including tumour formation. The latter is a major concern when using stem cells. The tumour forming ability is primarily due to their ability to proliferate for a long time and resist apoptosis. However, this is dependent on several factors such as donor’s age, patient’s health and MSC reaction at the target site. One approach to tackle this is to use MSC extracellular vesicles instead of whole cells. These vesicles contain therapeutic paracrine factors including mRNA, DNA and growth factors and have been found to mediate repair [23].

Regardless, the remarkable ability of MSCs (and stem cells in general) to differentiate into multiple cell types accompanied by advantages such as ease of isolation and expansion in-vitro, and the ability to extract these cells from multiple sources collectively make these cells lucrative options for therapeutic applications. MSCs are considered safe to use, but continuous follow-up studies through pre-clinical and clinical trials are necessary to ascertain this. Setting up more pre-clinical trials in small animals would be one step forward. Another step would be to use CRISPR/Cas9 technology for gene editing. The usage of this technology has shown promising results in various degenerative diseases [160] and can now be more frequently applied than before in pre-clinical trials. In the past, a large proportion of MSC studies focussed on and explored their osteogenic, chondrogenic and adipogenic potential aiming to ameliorate various conditions. Success has been achieved with this, for example, in ameliorating knee osteoarthritis and spinal cord injury. In recent years, the usage of MSCs in cancer therapy, particularly as a means of drug delivery has emerged and this approach has shown promising results in animal models [161–166].

### Generic Issues with Post-Transplant Cell Tracking

There are some generic problems associated with post-transplantation tracking following cell-based therapies. Utilising histopathology might be the gold standard for preclinical studies involving small animals, but it is labour intensive, time-consuming, and prone to errors and bias. For studies in humans, biopsy-driven tissue histopathology is neither always possible nor advisable or necessary, particularly, with the advent of non-invasive approaches that show successful in-vivo cell imaging. Another issue relates to the imaging approach itself. There are several imaging modalities that facilitate in-vivo imaging, for example, MRI, bioluminescence imaging, reporter gene labelling via fluorescence, fluorescent dyes, ultrasound, quantum dots and single-photon emission computed tomography (SPECT) and positron emission tomography (PET). However, all these present some limitations. For example, PET and MRI are more expensive than others and there is variability in sensitivity [167]. There are challenges in using the methods used too. Cells can be difficult to track with optical imaging if there has been direct labelling with fluorescent probes because the signal decreases in the tissue with time. It is even more challenging when the tissue is located deep within the body. Thus, long-term cell monitoring can be difficult. Radionuclides are high-energy particles that are less prone to attenuation in tissues. A combination of radionuclide-labelling and PET imaging is often used in clinical settings, but it can detect both dead and live cells giving an incorrect account of viable cells. MRI certainly offers an advantage by allowing MSC visualisation immediately after transplantation but there are issues such as signal dilution over time and the signal may not necessarily match with cell viability of transplanted cells due to non-specific particle uptake and signal retention by dead cells [168].

### MSC-Related Issues with Post-Transplant Cell Tracking

Some studies have shown issues related to labelling and tracking of MSCs. For example, MSC labelling with silica-coated gold nanorods impacted their cytokine profile [169] and MSC labelling with radioisotopes reduced MSC metabolic activity and migration [170]. Labelling can be achieved by transfection with a reporter gene, but over time the gene may be silenced leading to reductions in signal and product of the reporter gene. This can be misinterpreted as cell death. Indeed, constitutive mammalian promoters (ubiquitin and β-actin) are being utilised because these are believed to be more resistant to gene silencing. This approach has shown success [171]. However, there are concerns over reporter genes being integrated at random sites in the genome and this may alter MSC characteristics. Again, the aim is to counteract this by using editing technologies that allow site-specific DNA integration [168].

### The Concept of IONP-Labelling

Iron labelling of cells enables the usage of non-invasive MRI for cell detection and tracking, which imparts a huge advantage. Moreover, IONPs offer advantage over gadolinium in that iron is naturally present in the human body (about 3–4 g in an adult). With IONP-based MRI tracking, the dose is about 10 mg [25]. In theory, the body should be able to accommodate this subtle level of change in iron levels without causing major undesirable physiological outcomes. Iron oxide labelling of neural stem cells do cause alterations in the expression of some genes, including some iron-related genes [172] but these are believed to be temporary. Whether these alterations occur in humans too, and if so, whether these are temporary or permanent, needs to be investigated.

As in January 2022, a total of less than twenty clinical trials under ‘iron oxide nanoparticle’ are found on Clinical Trials Registers for Europe and US. While the first-generation IONPs like ferumoxide (Feridex and/or Endorem) and ferucarbotran (Resovist) are not used anymore in clinical applications in US and Europe, some of the currently registered trials use the 2nd generation of IONPs like ferumoxytol (Feraheme) and ferumoxtran-10. Since these do not show a great ability to be internalised by the cells, transfection agents are being used to achieve better cellular uptake. But usage of transfection agent is not problem-free (Table 3). Characteristic issues with its usage are nanoparticle precipitation due to mixing of negatively charged iron-oxides with positively charged transfection agent, and the need to use serum-free media to maximise the association between transfection agent and nanoparticles. However serum-free media cannot be used with all cells [25]. To tackle this, transfection agent-free approaches have been developed (Table 3).

Nevertheless, several studies have shown success in post-transplant monitoring of IONP-labelled cells in humans. For example, systemic delivery of IONP-labelled peripheral blood mononuclear cells was found to be safe. Healthy recipients showed no significant alterations in blood chemistry and cardiovascular physiology. The labelling did not alter cellular cytokine profile, migration, and homing, and the labelled cells were detectable via MRI for at least one week [173]. Also, IONP-labelling followed by MRI-based detection has been able to track transplanted cells in the spinal cord, occipital horns of multiple sclerosis patients and the brain of a child with ischemic brain injury [25].

## IONP-Labelling of MSCs

In the context of using IONPs on MSCs, some studies have shown inhibition of chondrogenic differentiation of MSCs following IONP-labelling (Feridex or Reovist) [174, 175]. Whether this would occur in humans needs to be investigated. One way to investigate the effect of IONP loading on MSCs is to ascertain endogenous ferritin levels in MSCs from different sources and determine the range of ferritin increment following IONP-labelling. This is particularly relevant when using iron oxide for labelling MSCs. While iron is necessary for cellular functions, excess iron is toxic and can feed into the Fenton reaction to generate reactive oxygen species. If in excess, these can cause cellular damage. Ferritin is the iron storage protein that can accommodate large amounts of iron ions and thereby reduce the possibility of excess-iron-induced damage. Examining the basal and IONP-induced ferritin levels in MSCs from different sources would better inform the IONP dosage to be used. Optimal IONP dosage would prevent excess-iron-induced damage to MSCs and help retain MSC viability and characteristics so that the cells function optimally following transplantation. This needs to be tested for MSCs from all sources because their iron-holding capacities may differ.

Generic concerns of cell-based therapy remain. These fundamentally relate to obtaining enough cells for transplantation, retaining their functional characteristics during in-vitro expansion, cell delivery to the target organ without viral or prion transmission, viability, differentiation, and functionality of cells upon reaching the target organ, immune response due to allogenic cell transplants, and carcinogenesis at the injection site. Questions that are yet to be answered include whether these cell-based therapies can improve the clinical outcome of all patients, whether this approach is applicable to all conditions, if yes, then to what extent, and if no, then to which conditions is the approach most and least applicable.

## Data Availability

Not applicable.

## Abbreviations

ALP: Alkaline phosphatase
BM-MSCs: Bone marrow-derived mesenchymal stem cells
BMP: Bone morphogenetic protein
CCL21: C-C motif ligand-21
CCR: C-C motif chemokine receptor
CXCR: C-X-C motif chemokine receptor
EGF: Epidermal growth factor
EGFR: Epidermal growth factor receptor
FGF-2: Fibroblast growth factor-2
GFP: Green fluorescent protein
HGF: Hepatocyte growth factor
HIF1-α: Hypoxia inducible factor 1 alpha
IL: Interleukin
IONPs: Iron-oxide nanoparticles
MCP-1: Monocyte chemoattractant protein-1
MRI: Magnetic resonance imaging
MSCs: Mesenchymal stem cells
PPARγ: Peroxisome proliferator-activated receptor gamma
SDF-1: Stromal cell-derived factor-1
SPIONPs: superparamagnetic iron oxide nanoparticles
TGF-β: Transforming growth factor beta
TNF-α: Tumour necrosis factor alpha
USSPIONP: Ultrasmall superparamagnetic iron oxide nanoparticles
VEGF: Vascular endothelial growth factor
αSMA: alpha smooth muscle actin
